# ﻿Phylogenomic and morphological evidence reveal a new species of spider lily, *Lycorislongifolia* (Amaryllidaceae) from China

**DOI:** 10.3897/phytokeys.210.90391

**Published:** 2022-10-05

**Authors:** Yi-Lei Lou, Dai-Kun Ma, Ze-Tao Jin, Hui Wang, Lu-Huan Lou, Shui-Hu Jin, Kun Liu, Bin-Bin Liu

**Affiliations:** 1 College of Landscape Architecture, Zhejiang Agriculture and Forestry University, Hangzhou 311300, China; 2 College of Landscape Architecture, Beijing Forestry University, Beijing 100083, China; 3 State Key Laboratory of Systematic and Evolutionary Botany, Institute of Botany, Chinese Academy of Sciences, Beijing 100093, China; 4 Centre of Pear Engineering Technology Research, State Key Laboratory of Crop Genetics and Germplasm Enhancement, Nanjing Agricultural University, Nanjing 210095, China; 5 College of Forestry and Biotechnology, Zhejiang Agriculture and Forestry University, Hangzhou 311300, China; 6 Anhui Provincial Key Laboratory of the Conservation and Exploitation of Biological Resources, College of Life Sciences, Anhui Normal University, Wuhu 241000, China

**Keywords:** *Lycoris*, morphological, phylogenomics, whole plastome

## Abstract

*Lycorislongifolia*, a new species from China, was described and illustrated here. Our phylogenomic evidence based on whole plastomes strongly supported the separate phylogenetic position of this new species, and morphologically it could also be distinguished by its long leaves with a distinct purplish-red midrib on the abaxial surface.

## ﻿Introduction

The genus *Lycoris* Herb., including ca. 13–20 species of flowering plants in the family Amaryllidaceae, subfamily Amaryllidoideae, is native to eastern and southern Asia. Herbert described the first species in 1820, *L.aurea* (L’Hér.) Herb., which has important ornamental and medicinal values (Hsu et al. 1994). In the mid-20^th^ century, an American horticulturist, Hayward, did much work on introducing and cultivating *Lycoris* species. Given the easily distinguished habit of the populations of *Lycorisaurea* distributed in northern Taiwan and southernmost Japan, i.e., the leaves appear in autumn, about a month later than *L.aurea*, and no remains of leaf bases (Hsu et al. 1994), Hayward described these populations as a new species, *L.traubii* W.Hayw. (Hayward 1957; Hsu et al. 1994; Kurita 1987). Having narrower perianth lobes and long-exserted stamens (Hsu et al. 1994; Ji and Meerow 2000), the populations from South Gansu (Kang Xian) and Northwest Hubei (Feng Xian) were described as a variety of *Lycorisaurea*, as L.aureavar.angustitepala P.S.Hsu, Kurita, Z.Z.Yu & J.Z.Lin (Hsu et al. 1994). In the last decades, numerous new species or hybrids of *Lycoris* have been published in its diversity center, i.e. mainland China, such as *L.hunanensis* M.H.Quan, L.J.Ou & C.W.She (Quan et al. 2013), *L.×hubeiensis* KunLiu (Meng et al. 2018), *L.tsinlingensis* P.C.Zhang, YiJunLu & TingWang (Lu et al. 2020), and *L.wulingensis* S.Y.Zhang (Zhang et al. 2021). Nowadays, more than 30 species and varieties have been recognized in the genus (Hsu et al. 1994; Ji and Meerow 2000; Kim 2004; Quan et al. 2013; Meng et al. 2018; Lu et al. 2020; Zhang et al. 2021), and nearly 20 of them are from China.

During our recent field explorations in Sichuan Province, China, we collected a wild flowering plant of *Lycoris*, which resembles *L.aurea* with yellow flowers. However, it could be easily distinguished from *L.aurea* by markedly long leaves with a distinct purplish-red midrib on the abaxial surface. Our morphological and molecular evidence strongly supported this population as a new *Lycoris* species.

## ﻿Materials and methods

Total genomic DNAs were extracted from 15mg of silica gel dried leaves using a modified CTAB method (Li et al. 2013). The library was prepared at the Molecular Biology Experiment Center, Germplasm Bank of Wild Species in Southwest China using a NEBNext Ultra^TM^ II DNA Library Prep Kit (New England Biolabs, Ipswich, MA, USA). The paired-end (150 bp) reads have been generated on the HiSeq 2500 (Illumina, Inc., San Diego, CA, USA) platform in Beijing Genomics Institution (BGI) (Shenzhen, China), ca. 8 GB of raw data for this new species. The raw reads have been deposited in the NCBI Sequence Read Archive in the BioProject (PRJNA857321) with the Run number SRR20072320.

The raw data generated from the Illumina platform was trimmed by Trimmomatic v.0.40 (Bolger et al. 2014) with the default parameters. The clean data was checked by FastQC (Andrews 2010) for quality control. We used the successive assembly approach (Zhang et al. 2015), combining the reference-based and the de novo assembly methods to assemble the chloroplast genome; this method has been performed well in various angiosperm lineages (e.g., Liu et al. 2019, 2020a, 2020b, 2021, 2022; Wang et al. 2020). We annotated the assembled chloroplast genome with two reference genomes (MK353216 and MH118290) downloaded from GenBank, and checked the start and stop codons carefully by translating the coding sequences of plastome into proteins in Geneious Prime (Kearse et al. 2012). We also verified the boundary of two reverse complementary repeats in the plastome using Find Repeats embedded in Geneious Prime (Kearse et al. 2012). The assembled chloroplast genome has been submitted to GenBank with the accession number ON960856. The gene map of the new species *Lycorislongifolia* chloroplast genome was drawn by OrganellarGenomeDRAW (OGDRAW) version 1.3.1 (Greiner et al. 2019).

We downloaded 24 chloroplast genomes from GenBank as the ingroup and *Narcissuspoeticus* L. as the outgroup for phylogenomic analysis. Given the potential effect of the missing data for the accurate phylogenetic inference, we used the whole plastome (WP) and 78 coding sequences (CDS) to estimate the phylogeny, respectively. Because of the nearly identical sequence of two inverted repeats (IR) in plastomes, we only included one repeat of IR region for downstream WP analyses. Each CDS sequence was extracted separately by Geneious Prime; the WP matrix was aligned with MAFFT v. 7.480 (Nakamura et al. 2018) with default parameters. The WP matrix was trimmed using trimAL v1.2 (Capella-Gutiérrez et al. 2009) with a heuristic method to decide on the best-automated method. All 78 CDS sequences of each plastome were concatenated by AMAS (Borowiec 2016). The best-fit partitioning schemes and/or nucleotide substitution models for the 78 CDS sequences were estimated using PartitionFinder2 (Stamatakis 2006; Lanfear et al. 2016), under the corrected Akaike information criterion (AICc) and linked branch lengths, as well as with rcluster (Lanfear et al. 2014) algorithm options. The resulting optimal partitioning schemes and evolutionary model for each CDS sequence were applied for the following tree inference. We used IQ-TREE2 v. 2.1.3 (Minh et al. 2020) with 1000 SH-aLRT and the ultrafast bootstrap replicates and RAxML 8.2.12 (Stamatakis 2014) with GTRGAMMA model for each partition and clade support assessed with 200 rapid BS replicates for the Maximum Likelihood (ML) analysis. The BI was performed with MrBayes 3.2.7 (Ronquist et al. 2012). The Markov Chain Monte Carlo (MCMC) analyses were run for 10,000,000 generations. The stationarity was regarded to be reached when the average standard deviation of split frequencies remained below 0.01. Trees were sampled every 1,000 generations, and the first 25% of samples were discarded as burn-in. The remaining trees were used to build a 50% majority-rule consensus tree. Considering the possible different evolutionary forces in the chloroplast genome, we also used ASTRAL-III (Zhang et al. 2018) for estimating a coalescent-based species tree based on the 78 CDS sequences.

## ﻿Results

**Figure 1.**
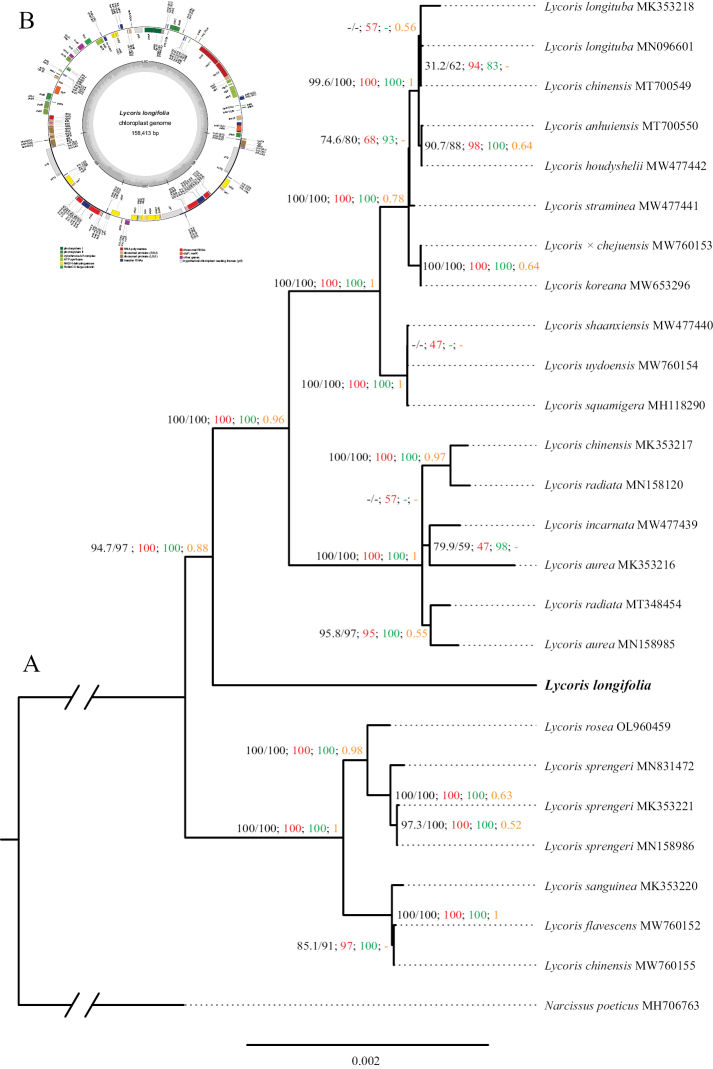
Maximum likelihood phylogeny of *Lycoris* inferred from RAxML analysis of the whole plastome data **A** numbers above the branches indicate the SH-aLRT support and Ultrafast Bootstrap support (black) by IQ-TREE2, the bootstrap support (red) by RAxML, the posterior probabilities (green) by MrBayes, and the local posterior possibility (orange) by ASTRAL-III. The upper-left inset was a gene map of the new species *Lycorislongifolia* chloroplast genome **B**.

The chloroplast genome of *Lycorislongifolia* was 158,413 bp in length, with a typical quadripartite structure consisting of a large single copy region and a small single copy region separated by two long inverted repeats (Fig. 1B). And this structure has been nearly similar to other *Lycoris* chloroplast genomes released in GenBank. They contained the same number of coding sequences (78), tRNAs (30), and rRNAs (4).

The WP matrix was 131,649 bp in length, with the poor sites trimmed by trimAL (Capella-Gutiérrez et al. 2009); the concatenated CDSs were 67,953 bp in length. These two matrices generated seven trees (Fig. 1A, Suppl. material 1–6). The four ML trees (Fig. 1A, Suppl. material 1, 3, 4), two Bayesian trees (Suppl. material 2, 5), and the species tree (Suppl. material 6) resulted in a consistent phylogenetic position, and this new species, *Lycorislongifolia*, formed a separate clade (Fig. 1A). This result showed that this new species has been distant from other species in *Lycoris*. The examined morphological characters, long leaves and purplish-red midrib abaxially, also supported its distinguished status.

### ﻿Taxonomy

#### ﻿ Lycoris longifolia

Taxon classificationPlantaeAsparagalesAmaryllidaceae

L.H.Lou sp. nov.

E86E39DC-CE8B-52F1-95CE-5F9705ED96F0

urn:lsid:ipni.org:names:77306298-1

**Figure 2.**
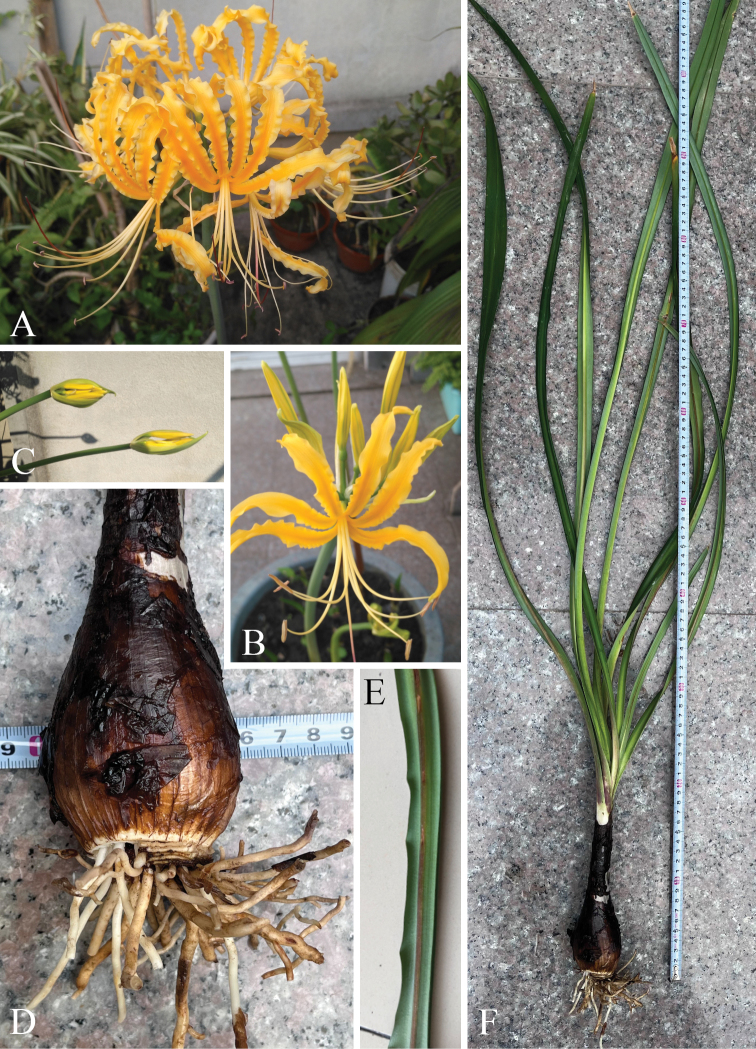
Field photos of *Lycorislongifolia***A-C** flowers **D** bulb **E** the distinct purplish-red midrib abaxially **F** vegetative growth period, showing the long leaves.

**Figure 3.**
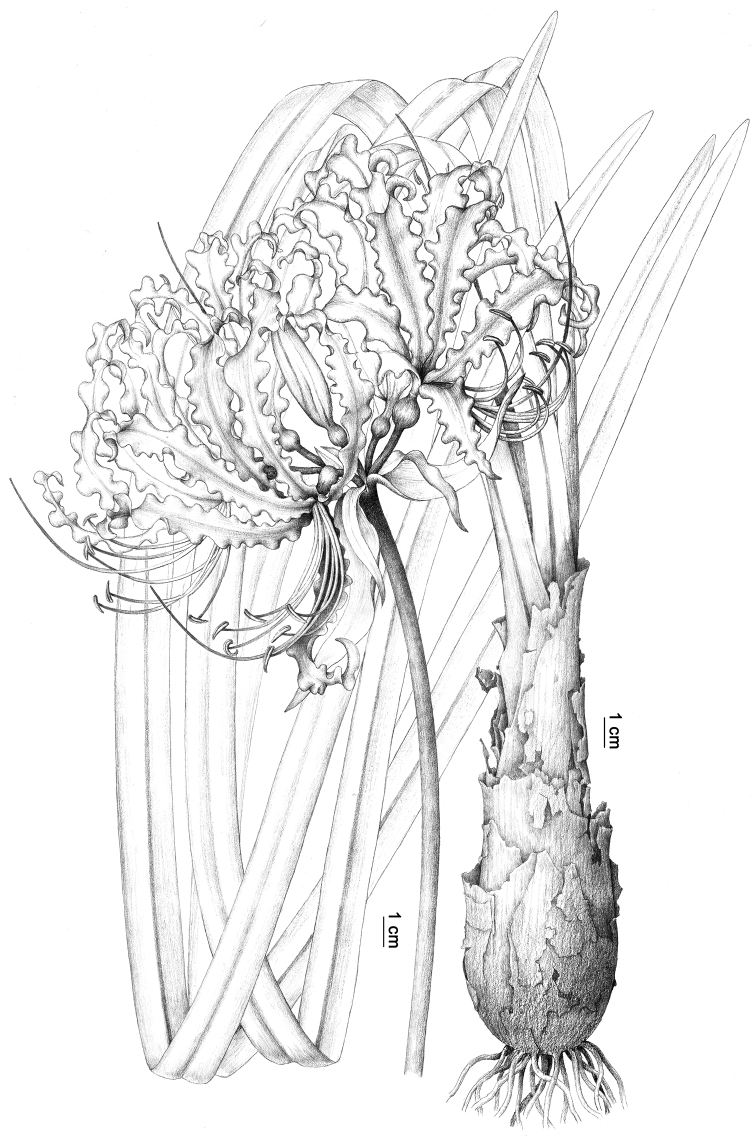
Illustration of *Lycorislongifolia*, drawn by Ai-Li Li (PE).

Chinese name: 长叶石蒜 Figs 2 , 3

##### Diagnosis.

Most similar to *L.aurea* but differs from it by markedly longer leaves, abaxially with a distinct purplish-red midrib.

##### Type.

China. Sichuan: Ya’an, Yucheng, Bifengxia, Houyancun, Yanjiashan, under the shrub along the stream, elevation ca. 950 m, 10 May 2021, *L.H. Lou & Y.L. Lou 8765* (holotype PE [barcode 02347459]!; isotypes KUN!, PE [barcode 02347457]!).

##### Additional Specimens examined.

China. Sichuan: Ya’an, Yucheng, Bifengxia, Houyancun, Yanjiashan, under the shrub along the stream, elevation ca. 950 m, 30 July 2021, *L.H. Lou & Y.L. Lou 8766* (paratype PE [barcode 02347458]!).

##### Description.

Bulbous perennial. Bulbs subglobose, 3–6 cm diam., tunics membranous, dark brown. Leaves ligulate, acute at the apex, ca. 80–120 × 1.5–2 cm, absent at the flowering time and appearing in autumn, dark green, with a prominent midrib on the abaxial surface, abaxial midrib distinctly purplish-red. Inflorescence scapose, umbellate; scape solid, 70–75 cm long, ca. 2.0 cm diam. at base, light green with purplish-red base; involucral bracts 2, lanceolate, 5.0–9.0 cm long by 1.8 cm wide at base, membranous, light green; bracteoles membranous, lanceolate, 1.0–4.0 cm long. Flowers 5–7 per umbel; pedicels 2–2.5 cm long; perianth with 6 tepals; tube ca. 1.5 cm; lobes yellow, abaxially with white mid-vein, strongly recurved, narrowly oblanceolate, ca. 7 × 0.8–1.0 cm, margin strongly undulate. Stamen filaments 6, creamy-yellow, slightly longer than perianth; anther light purplish, dorsifixed, 8–10 mm long before anthesis. Style creamy-yellow but rose-red at apex, slightly exceeding filaments; stigma purplish-red; ovary green, ovoid, ca. 5 mm long.

##### Phenology.

Scape produced from July to August, and vegetative growth from September to May next year. This new species grows along the forest edge near the riverside, and *Quercusglauca* Thunb. and *Pinusmassoniana* Lamb. are the dominant associated species.

##### Etymology.

The specific epithet alludes to length of leaf blades, a diagnostic character.

##### Distribution.

This new species has been narrowly discovered in Ya’an, Sichuan, China. Some localities of Southwestern China have been poorly discovered, and a comprehensive floristic investigation will help elucidate the germplasm resources.

### ﻿Key to the species of *Lycoris* in China

**Table:** 

1	Flowers actinomorphic	**2**
–	Flowers zygomorphic	**6**
2	Margin of perianth lobes not undulate	**3**
–	Margin of perianth lobes basally minutely undulate	**4**
3	Perianth pale purple but apically blue	***L.sprengeri***
–	Perianth white or yellow	***L.longituba***
4	Perianth purple	***L.squamigera***
–	Perianth not purple	**5**
5	Perianth yellow	***L.anhuiensis***
–	Perianth white, abaxially with purple midvein	***L.incarnata***
6	Leaves appearing in autumn	**7**
–	Leaves appearing in spring	**15**
7	Perianth yellow or ocher-yellow	**8**
–	Perianth bright red, deep red, rose-red, or white	**11**
8	Perianth yellow; leaves 1.5–5 cm wide	**9**
–	Perianth ocher-yellow; leaves 1.0–1.5 cm wide	**10**
9	Leaves ensiform, ca. 60 × 2–5 cm	***L.aurea***
–	Leaves ligulate, ca. 100 × 1.5–2 cm	***L.longifolia***
10	Leaves ensiform, apex acuminate	***L.straminea***
–	Leaves ligulate, apex obtuse	***L.hunanensis***
11	Perianth bright red, deep red, or rose-red	**12**
–	Perianth white	***L.houdyshelii***
12	Perianth bright red or deep red, lobes strongly recurved	**13**
–	Perianth rose-red, lobes slightly recurved	**14**
13	Perianth bright red	***L.radiata***
–	Perianth deep red with white but faintly pale red filaments	***L.hubeiensis***
14	Leaves ligulate, ca. 0.8 cm wide	***L.rosea***
–	Leaves narrowly ligulate, ca. 0.5 cm wide	***L.wulingensis***
15	Perianth white	**16**
–	Perianth yellow or orange-red	**17**
16	Perianth white without pink stripes	***L.caldwellii***
–	Perianth white with pink stripes	***L.shaanxiensis***
17	Perianth yellow in bud, becoming orange-red as buds develop	***L.tsinlingensis***
–	Perianth yellow	**18**
18	Perianth lobes without red stripes	***L.chinensis***
–	Perianth lobes abaxially with red stripes	***L.guangxiensis***

## Supplementary Material

XML Treatment for Lycoris longifolia

## Supplementary materials

Supplementary material 1 Figure S1 Data type Images. Explanation note Maximum likelihood phylogeny of *Lycoris* inferred from IQ-TREE2 analysis of the whole plastome data. Numbers above the branches indicate the SH-aLRT support and Ultrafast Bootstrap support. https://binary.pensoft.net/file/753069This dataset is made available under the Open Database License (http://opendatacommons.org/licenses/odbl/1.0/). The Open Database License (ODbL) is a license agreement intended to allow users to freely share, modify, and use this Dataset while maintaining this same freedom for others, provided that the original source and author(s) are credited.Yi-Lei Lou, Dai-Kun Ma, Ze-Tao Jin, Hui Wang, Lu-Huan Lou, Shui-Hu Jin, Kun Liu, Bin-Bin Liu

Supplementary material 2 Figure S2 Data type Images. Explanation note Bayesian inference phylogeny of *Lycoris* inferred from MrBayes analysis of the complete chloroplast genome data. Numbers above the branches indicate the posterior probabilities (PP). https://binary.pensoft.net/file/753070This dataset is made available under the Open Database License (http://opendatacommons.org/licenses/odbl/1.0/). The Open Database License (ODbL) is a license agreement intended to allow users to freely share, modify, and use this Dataset while maintaining this same freedom for others, provided that the original source and author(s) are credited.Yi-Lei Lou, Dai-Kun Ma, Ze-Tao Jin, Hui Wang, Lu-Huan Lou, Shui-Hu Jin, Kun Liu, Bin-Bin Liu

Supplementary material 3 Figure S3 Data type Images. Explanation note Maximum likelihood phylogeny of *Lycoris* inferred from IQ-TREE2 analysis of the concatenated 78 plastid coding genes. Numbers above the branches indicate the SH-aLRT support and Ultrafast Bootstrap support. https://binary.pensoft.net/file/753071This dataset is made available under the Open Database License (http://opendatacommons.org/licenses/odbl/1.0/). The Open Database License (ODbL) is a license agreement intended to allow users to freely share, modify, and use this Dataset while maintaining this same freedom for others, provided that the original source and author(s) are credited.Yi-Lei Lou, Dai-Kun Ma, Ze-Tao Jin, Hui Wang, Lu-Huan Lou, Shui-Hu Jin, Kun Liu, Bin-Bin Liu

Supplementary material 4 Figure S4 Data type Images. Explanation note Maximum likelihood phylogeny of *Lycoris* inferred from RAxML analysis of the concatenated 78 plastid coding genes. Numbers above the branches indicate the bootstrap support. https://binary.pensoft.net/file/753072This dataset is made available under the Open Database License (http://opendatacommons.org/licenses/odbl/1.0/). The Open Database License (ODbL) is a license agreement intended to allow users to freely share, modify, and use this Dataset while maintaining this same freedom for others, provided that the original source and author(s) are credited.Yi-Lei Lou, Dai-Kun Ma, Ze-Tao Jin, Hui Wang, Lu-Huan Lou, Shui-Hu Jin, Kun Liu, Bin-Bin Liu

Supplementary material 5 Figure S5 Data type Images. Explanation note Bayesian inference phylogeny of *Lycoris* inferred from MrBayes analysis of the complete chloroplast genome data. Numbers above the branches indicate the posterior probabilities (PP). https://binary.pensoft.net/file/753073This dataset is made available under the Open Database License (http://opendatacommons.org/licenses/odbl/1.0/). The Open Database License (ODbL) is a license agreement intended to allow users to freely share, modify, and use this Dataset while maintaining this same freedom for others, provided that the original source and author(s) are credited.Yi-Lei Lou, Dai-Kun Ma, Ze-Tao Jin, Hui Wang, Lu-Huan Lou, Shui-Hu Jin, Kun Liu, Bin-Bin Liu

Supplementary material 6 Figure S6 Data type Images. Explanation note Species tree of *Lycoris* inferred from ASTRAL-III of the 78 plastid coding genes. Numbers above the branches indicate the branch support values measuring the support for a local posterior possibility. https://binary.pensoft.net/file/753074This dataset is made available under the Open Database License (http://opendatacommons.org/licenses/odbl/1.0/). The Open Database License (ODbL) is a license agreement intended to allow users to freely share, modify, and use this Dataset while maintaining this same freedom for others, provided that the original source and author(s) are credited.Yi-Lei Lou, Dai-Kun Ma, Ze-Tao Jin, Hui Wang, Lu-Huan Lou, Shui-Hu Jin, Kun Liu, Bin-Bin Liu
